# Effect of egg yolk powder on freezability of Murrah buffalo (*Bubalus bubalis*) semen

**DOI:** 10.14202/vetworld.2016.601-604

**Published:** 2016-06-17

**Authors:** N. Kumar, S. A. Lone, J. K. Prasad, M. H. Jan, S. K. Ghosh

**Affiliations:** 1 Division of Animal Reproduction, Gynecology & Obstetrics, NDRI, Karnal, Haryana, India; 2 Germ Plasm Centre, Division of Animal Reproduction, Indian Veterinary Research Institute, Izatnagar, Bareilly, Uttar Pradesh, India; 3 Central Institute for Research on Buffalo, Hisar, Haryana, India

**Keywords:** buffalo semen, egg yolk powder, freezability

## Abstract

**Aim:**

The aim of this study was to investigate the effect of commercial egg yolk powder as an alternative to fresh egg yolk on freezability of Murrah buffalo semen.

**Materials and Methods:**

Semen samples (12) from 3 Murrah buffaloes (4 from each bull) with mass motility (≥3+) and total motility (70% and above) were utilized in this study. Immediately after collection, each sample was divided into four groups. Groups I was diluted up to 60×10^6^ sperm/ml with tris extender containing 10% fresh egg yolk and Groups II, III, and IV were diluted up to 60×10^6^ sperm/ml with tris extender containing 2%, 4%, and 6% egg yolk powder, respectively. Semen samples were processed and cryopreserved followed by examination of frozen semen samples after 24 h. Semen samples from each group were evaluated for total motility, viability, acrosomal integrity, abnormality, and hypo-osmotic swelling test (HOST) response after dilution, pre-freeze, and post-thaw stage.

**Results:**

Pre-freeze total motility was significantly (p<0.05) higher in Groups III and IV as compared to Groups I and II, and post-thaw total motility was significantly (p<0.01) higher in Group III as compared to other three groups. Viability was significantly (p<0.05) higher in Groups II, III, and IV than Group I at the pre-freeze stage. Significantly (p<0.01) higher viability and acrosomal integrity were recorded in Group III as compared to other three groups at the post-thaw stage. Abnormality was significantly (p<0.05) higher in Group IV than other three groups. HOST response was significantly (p<0.05) higher in Groups II and III than Groups I and IV at the pre-freeze and post-thaw stages.

**Conclusion:**

Addition of egg yolk powder at 4% level yielded significantly better results in terms of post-thaw semen quality as compared to the fresh egg yolk and other concentrations of egg yolk powder (2% and 6%).

## Introduction

The beneficial effect of egg yolk on spermatozoa during liquid storage and cryopreservation was first time reported by Phillips and Lardy in 1940 [1]. Egg yolk protects spermatozoa against cold shock, reduces loss of acrosomal enzymes, and helps in preserving semen quality [2]. The protective effects of egg yolk are due to low-density lipoprotein fraction present in it. Egg yolk has been included in dilutor at concentrations ranging from 1.5% to 50% [2]. Harvesting egg yolk is a cumbersome process because it involves proper disease screening, disinfection, skilful breaking of the outer shell, and inner membrane to separate the yolk from albumin and chalazae [3].

Due to the animal origin of egg yolk, it represents a potential risk of microbial contamination in the diluent. Although egg yolk benefits spermatozoa during cryopreservation, it can represent a potential risk to cells as they may contain specific microbial agents or other contaminants that may compromise sperm quality [4]. Terrestrial Animal Health Code (OIE) recommended that products used to treat spermatozoa should originate from sources of animal origin, which are free from any health risks [5]. This can be accomplished using egg yolk powder, instead of fresh egg yolk, as it is pasteurized. The use of egg yolk powder is documented for the cryopreservation of ovine [6], Zebu bull [7], and buffalo [8] and it is reported that semen treated with egg yolk powder had higher post-thaw motility as compared to fresh egg yolk treated semen.

In buffalo semen, egg yolk powder was used at the rate of 5%, 10%, and 20%, and it was shown that at higher concentration of egg yolk powder (20%), the post-thaw quality of semen deteriorated [8]. It is, therefore, hypothesized that the use of egg yolk powder in the dilutor for the cryopreservation of buffalo bull semen at lesser concentration as reported earlier will not compromise the post-thaw attributes, and the same will reduce the cost of cryopreservation. Hence, the present study was designed to investigate the effect of egg yolk powder on quality and freezability of buffalo semen.

## Materials and Methods

### Ethical approval

No ethical approval was necessary to pursue this research work.

### Experimental animals

Three healthy breeding buffalo bulls of approximately 4-6 years of age maintained at GermPlasm Center of Animal Reproduction Division, IVRI, Izatnagar, were utilized for the study. These bulls were kept under identical feeding and managemental conditions during the entire course of theinvestigation.

### Collection of semen and its processing

Four different dilutors containing 2%, 4%, and 6% w/v egg yolk powder (Sigma, USA) and 10% v/v fresh egg yolk, respectively, were prepared and kept at 37°C. A total of 12 ejaculates (4 from each bull) were collected from three Murrah breeding bulls with artificial vagina. Ejaculates with mass motility ≥3+ and total motility 70% and above were utilized for the present study. Semen ejaculates that qualified for cryopreservation were split into four groups. Group I was diluted with tris-egg yolk-glycerol dilutor (containing 10% fresh egg yolk) up to 60×10^6^ spermatozoa/ml. Groups II, III, and IV were diluted with tris dilutor containing 2%, 4%, and 6% egg yolk powder, respectively. After dilution, the percentage of various seminal attributes (total motility, viability, acrosomal integrity, abnormality, and hypo-osmotic swelling response) was recorded in each group.

### Semen freezing and its evaluation

French mini straws (0.25 ml) were filled with the diluted semen samples, sealed with polyvinyl alcohol powder and kept for 4 h at 5°C for equilibration. After equilibration, straws were kept in automatic programmable biological cell freezer (IMV Technology, France) until the temperature of straws reached −140°C. Then, straws were plunged into liquid nitrogen (−196°C) for storage and stored for 24 h before thawing at 37°C for 30 s. From each group, semen samples were evaluated for various seminal attributes (total motility, viability, acrosomal integrity, abnormality, and hypo-osmotic swelling response) at pre-freeze and post-thaw stage.

### Semen evaluation

#### Seminal attributes

A drop of the diluted semen was kept on a clean, grease free, pre-warmed glass slide; cover slip was placed, and total motility was assessed under high power magnification (400×magnification) of a phase contrast microscope. Percentage of live and abnormal spermatozoa was determined by a differential staining technique using Eosin-Nigrosin stain [9]. Acrosomal intactness was determined by Giemsa stain as per method described by Watson [10]. Hypo-osmotic swelling test (HOST) was carried out according to the method described by Jeyendran *et al*. [11].

### Statistical analysis

Data were statistically analyzed by one-way ANOVA and results were expressed as mean±standard error. Means were compared using Tukey’s multiple comparisons test. The statistical package of GraphPad Prism, San Diego, USA was used for analyzing the data.

## Results and Discussion

### Total motility

The mean values of total motility are presented in Table-1. The total motility of a semen sample gives a good indication of fertility of the bull and ability of spermatozoa to withstand the stress of the cryopreservation process. After dilution, the percentage of total motility was 74.67±1.09, 74.50±0.92, 74.58±1.03, and 75.17±1.15 in Groups I, II, III, and IV, respectively. No significant difference in motility percentage at post-dilution was recorded among any groups. After dilution, the percentage of motile spermatozoa was higher than the values reported by Mittal *et al*. [12]. At pre-freeze stage, the percentage of motile spermatozoa was significantly (p<0.05) higher in Groups III and IV as compared to Groups I and II. At pre-freeze stage, the percentage of motile spermatozoa was higher than the values reported by Rao *et al*. [13]. At post-thaw stage, percentage of individual motility was significantly (p<0.01) higher in Group III as compared to other three groups. The percentage reduction in total motility was 35.48, 33.08, 26.18, and 35.14 in Groups I, II, III, and IV, respectively.

**Table-1:** Effect of egg yolk powder on seminal attributes and sperm function of buffalo semen at post-dilution, pre-freeze, and post-thaw stage.

Seminal attributes	Stage	Group I	Group II	Group III	Group IV
Total motility	PD	74.67±1.09	74.50±0.92	74.58±1.03	75.17±1.15
	PF	67.42±1.19^B^	67.78±0.99^B^	69.50±1.14^A^	68.92±1.16^A^
	PT	48.17±0.71^b^	49.85±0.78^b^	54.58±0.65^a^	48.75±0.83^b^
Viability	PD	76.25±1.07	76.25±1.28	77.17±1.22	76.83±1.17
	PF	69.67±1.19^B^	70.75±1.19^A^	71.08±1.31^A^	70.17±1.22^A^
	PT	54.78±1.44^b^	55.34±0.75^b^	61.33±0.71^a^	54.25±1.00^b^
Acrosomal integrity	PD	78.58±1.01	78.50±1.13	78.83±1.15	78.42±1.15
	PF	72.50±1.00	72.75±1.21	72.67±1.10	72.83±1.01
	PT	57.69±0.88^b^	57.50±0.71^b^	65.67±0.54^a^	58.50±0.71^b^
Abnormality	PD	10.33±0.47	10.33±0.45	9.83±0.41	10.67±0.41
	PF	11.00±0.46	10.75±0.41	10.58±0.38	11.17±0.46
	PT	11.25±0.45^B^	11.25±0.48^B^	10.78±0.40^B^	12.50±0.50^A^
HOS (%)	PD	68.75±0.87	69.83±0.74	69.33±0.78	69.75±0.81
	PF	61.08±0.81^B^	63.17±0.81^A^	62.67±0.69^A^	60.92±0.82^B^
	PT	41.76±0.69^B^	44.17±0.79^A^	45.50±0.94^A^	40.83±0.72^B^

PD=Post-dilution, PF=Pre-freeze, PT=Post-thaw. Groups I, II, III, and IV contain 10% fresh egg yolk, 2%, 4%, and 6% egg yolk powder, respectively. Mean showing different superscripts in upper case letters (A and B) and lower case letters (a and b) in row differ significantly at 5% (p<0.05) and 1% (p<0.01), respectively

### Viability

Viability of spermatozoa in a semen sample is significantly and positively correlated with initial motility, post-thaw motility, and fertility of spermatozoa. Post-dilution viability was 76.25±1.07, 76.25±1.28, 77.17±1.22, and 76.83±1.17 in Groups I, II, III, and IV, respectively. The percentage of live spermatozoa was higher than the values reported by Bhakat *et al*. [14]. No significant differences in viability percentage were recorded among any groups post-dilution. At pre-freeze stage, percent viability was significantly (p<0.05) higher in Groups II, III, and IV as compared to Group I. At post-thaw stage, percent viability was significantly (p<0.01) higher in Group III as compared to Groups I, II, and IV. The percentage reduction in viability was 28.15, 27.42, 20.52, and 29.61 in Groups I, II, III, and IV, respectively.

### Acrosomal integrity

Acrosomal integrity of mammalian spermatozoa is prerequisite for capacitation, normal acrosome reaction, and successful fertilization *in vivo*. The percentage of intact acrosomes after dilution in Groups I, II, III, and IV was 78.58±1.01, 78.50±1.13, 78.83±1.15, and 78.42±1.15, respectively. The percentage of spermatozoa with intact acrosome was higher than the values reported by Meena *et al*. [15] and Patel and Siddiquee [16]. After dilution and pre-freeze stage, no significant difference in the percentage of acrosomal integrity was recorded among any groups. However, at post-thaw stage, the percentage of spermatozoa with intact acrosome was significantly (p<0.01) higher in Group III as compared to other three groups. Values of acrosomal integrity in all groups were lower which can normally occur in buffalo semen and also observed by Singh *et al*. [8]. Percentage decline in acrosomal integrity was 26.58, 26.75, 16.69, and 25.40 in Groups I, II, III, and IV, respectively.

### Abnormality

After dilution, the percentage of abnormal spermatozoa in Groups I, II, III, and IV was 10.33±0.47, 10.33±0.45, 9.83±0.41, and 10.67±0.41, respectively. The percentage of abnormal spermatozoa was in agreement to the reports of Bhakat *et al*. [14]. No significant difference in the percentage of abnormal spermatozoa was recorded after dilution and at the pre-freeze stage. However, at post-thaw stage, the percentage of abnormal spermatozoa were significantly (p<0.05) higher in Group IV as compared to other three groups.

### HOST response

The percentage of HOST positive spermatozoa after dilution in Groups I, II, III, and IV were 68.75±0.87, 69.83±0.74, 69.33±0.78, and 69.75±0.81, respectively. The HOST (%) in our study was comparable to values of Meena *et al*. [15] but higher than the values reported by Bhakat *et al*. [14]. No significant difference in HOST response was recorded after dilution among any groups. However, at pre-freeze and post-thaw stage, the percentage of HOST positive spermatozoa were significantly (p<0.05) higher in Groups II and III as compared to Groups I and IV. The percentage reduction in HOST response was 39.25, 36.76, 34.37, and 41.46 in Groups I, II, III, and IV, respectively.

## Conclusion

Addition of egg yolk powder at 4% level in the extender yielded significantly better results in terms post-thaw semen quality as compared to the fresh egg yolk and other levels of egg yolk powder (2% and 6%). Egg yolk powder at a level of 4% can be effectively used as an alternative to fresh egg yolk for cryopreservation of Murrah buffalo semen.

## Author’s Contributions

NK and SAL planned and carried out research work. JKP and SKG provided lab facility and necessary help during research work. MHJ helped in the statistical analysis of Data. All authors read and approved the final manuscript.
